# The impact of air pollution on interstitial lung disease: a systematic review and meta-analysis

**DOI:** 10.3389/fmed.2023.1321038

**Published:** 2024-01-17

**Authors:** Doris Lan, Caitlin C. Fermoyle, Lauren K. Troy, Luke D. Knibbs, Tamera J. Corte

**Affiliations:** ^1^Central Clinical School, Faculty of Medicine and Health, The University of Sydney, Sydney, NSW, Australia; ^2^Department of Respiratory and Sleep Medicine, Royal Prince Alfred Hospital, Camperdown, NSW, Australia; ^3^National Health and Medical Research Council (NHMRC), Centre of Research Excellence in Pulmonary Fibrosis, Camperdown, NSW, Australia; ^4^School of Public Health, Faculty of Medicine and Health, The University of Sydney, Sydney, NSW, Australia; ^5^Public Health Unit, Public Health Research Analytics and Methods for Evidence (PHRAME), Sydney Local Health District, Camperdown, NSW, Australia

**Keywords:** air pollution, interstitial lung disease (ILD), idiopathic pulmonary fibrosis (IPF), ozone (O_3_), nitrogen dioxide (NO_2_), particulate matter (PM_2.5_, PM_10_), sulfur dioxide (SO_2_)

## Abstract

**Introduction:**

There is a growing body of evidence suggesting a causal relationship between interstitial lung disease (ILD) and air pollution, both for the development of the disease, and driving disease progression. We aim to provide a comprehensive literature review of the association between air pollution, and ILD, including idiopathic pulmonary fibrosis (IPF).

**Methods:**

We systematically searched from six online database. Two independent authors (DL and CF) selected studies and critically appraised the risk of bias using the Newcastle-Ottawa Scale (NOS). Findings are presented through a narrative synthesis and meta-analysis. Meta-analyses were performed exclusively when there was a minimum of three studies examining identical pollutant-health outcome pairs, all evaluating equivalent increments in pollutant concentration, using a random effects model.

**Results:**

24 observational studies conducted in 13 countries or regions were identified. Pollutants under investigation encompassed ozone (O_3_), nitrogen dioxide (NO_2_), Particulate matter with diameters of 10 micrometers or less (PM_10_) and 2.5 micrometers or less (PM_2.5_), sulfur dioxide (SO_2_), carbon monoxide (CO), nitric oxide (NO) and nitrogen oxides (NOx). We conducted meta-analyses to assess the estimated Risk Ratios (RRs) for acute exacerbations (AE)-IPF in relation to exposure to every 10 μg/m^3^ increment in air pollutant concentrations, including O_3_, NO_2_, PM_10_, and PM_2.5._ The meta-analysis revealed a significant association between the increased risk of AE-IPF in PM_2.5_, yielding RR 1.94 (95% CI 1.30–2.90; *p* = 0.001). Findings across all the included studies suggest that increased exposure to air pollutants may be linked to a range of health issues in individuals with ILDs.

**Conclusion:**

A scarcity of available studies on the air pollutants and ILD relationship underscores the imperative for further comprehensive research in this domain. The available data suggest that reducing levels of PM_2.5_ in the atmosphere could potentially reduce AE frequency and severity in ILD patients.

## 1 Introduction

Interstitial lung disease (ILD) refers to a group of chronic lung disorders (1) characterized by inflammation and fibrosis of the lung interstitium (2, 3) including idiopathic pulmonary fibrosis (IPF), sarcoidosis, hypersensitivity pneumonitis, and connective tissue disease associated ILD (CTD-ILD) (4). Symptoms vary, but commonly include breathlessness, cough, and fatigue (5–8), and these symptoms can significantly decrease quality of life (QoL) for individuals with ILD. Moreover, prevalent comorbid conditions including gastro-oesophageal reflux disease (GORD), obstructive sleep apnoea (OSA), and depression can further contribute to the negative impact on QoL (5).

Prognosis is highly variable amongst ILD patients, with some patients having very rapid progression, and others a more indolent disease course. IPF, in particular, is associated with a high symptom burden, significant comorbidities, and a shortened life expectancy (9–12), with a 5-year mortality rate of 69% when not receiving antifibrotic treatment (13).

Recognized risk factors for the development of ILD include advanced age, male sex, genetic factors, smoking, medications, systemic autoimmune diseases, and occupational or environmental exposures (14–22). In the past decade, the impact of geographic factors, specifically air pollution, on lung health has received growing attention. Air pollutants have been identified as triggers for exacerbations of COPD and are associated with worsened asthma symptoms (23, 24). These pollutants also play a role in increasing the likelihood of lung cancer in individuals at risk (23, 24). Furthermore, there is evidence indicating an elevated risk of respiratory infections (24, 25), including tuberculosis (25) associated with exposure to air pollutants.

Air pollution is a complex combination of solid particles, liquid droplets, and various gases (26). It originates from a wide array of sources, including household fuel combustion, industrial smokestacks, vehicle emissions, power generation, the open burning of waste, agricultural activities, desert dust, and numerous other origins (26). Common air pollutants measured include particulate matters, where particles with an aerodynamic diameter of equal or less than 2.5 micrometre (PM_2.5_) and particles with an aerodynamic diameter of 10 micrometre (PM_10_), ozone (O_3_), nitrogen dioxide (NO_2_), carbon monoxide (CO) and sulfur dioxide (SO_2_) (26).

There has been increasing interest in the relationship between air quality and ILD with most studies and review papers focusing on IPF. In this systematic review with meta-analysis, we evaluate the relationship between air pollution and the broader ILD population. By incorporating all ILD subgroups, we aim to provide a comprehensive overview of the association between various pollutants and health outcomes, and to inform the understanding of underlying mechanisms and the potential public health implications.

## 2 Methods

### 2.1 Review protocol

The review followed the PRISMA 2020 (Preferred Reporting Items for Systematic Reviews and Meta-Analyses) guideline (27). Given the specific focus on potential associations between exposure and clinical outcomes, instead of applying the conventional PICO (Population, Interventions, Comparators, and Outcomes) framework, the PEO (Patient, Exposure of interest, and Outcome) approach was employed (28). In this framework, the population of interest focused on two primary groups: those with a diagnosis of ILD and those who subsequently develop ILD. The exposure of interest is air pollution. The outcome examined is the impact of air pollution on ILD patients, including but not restricted to, acute exacerbation of ILD, disease progression, hospitalisation, and mortality rates associated with ILD and the risk of ILD development in the general population exposed to air pollution (Table 1). Our primary objective is to comprehensively explore the diverse impacts of air pollution on ILD. We anticipate encountering a spectrum of outcomes and surrogate markers beyond those explicitly specified, allowing for a thorough examination of this complex relationship.

**Table 1:** PEO worksheet (impact of air pollution on interstitial lung disease).

Population	Individuals with ILD or general population
Exposure of interest (independent variable)	Air pollution
Outcome (dependent variable)	Impact of air pollution on individuals with ILD, or risk of developing ILD in general population

### 2.2 Eligibility criteria

All authors engaged in thorough deliberation and reached a consensus regarding the inclusion and exclusion criteria. Inclusion criteria for this review were: (1) original research studies; (2) human studies; (3) studies examining the pollutants of interest in individuals diagnosed with ILD or in the general population who develop ILD. Exclusion criteria were: (1) studies exploring the impact of air pollution on general lung health and respiratory disease other than ILD; (2) studies focusing on other risk factors such as occupational factors in ILD; (3) studies measuring indoor exposure; and (4) qualitative studies.

### 2.3 Search strategy and information sources

Articles were searched for using the following six electronic databases: PubMed, Medline, Cochrane Library, CINAHL, Embase, and Scopus with latest search carried out on 20 July 2023. The key terms used were: (Air quality or air pollution or environment*) and (impact or outcome or relationship or effect or association) and (Pulmonary Fibrosis or interstitial lung disease or ILD). Key terms were searched as key words rather than subject headings to explore the topic as widely as possible. The search was then limited to the years 2000 to the present and “English Language”.

Apart from the electronic databases search, a snowball search technique (29) was used to find similar papers to the identified key papers. A secondary manual search of the reference lists was also carried out if the title suggested relevance to the searching terms. In addition, articles that are not able to be obtained from on-line library were requested through the Royal Prince Alfred Hospital library.

### 2.4 Study selection

Two independent authors (DL and CF) selected studies for inclusion. During the selection process, disagreements regarding the inclusion of specific articles meeting the predefined criteria were successfully resolved through discussion and consensus between the selecting authors. Studies lacking individual-level data, such as ecological studies, were considered for inclusion in the review but were ultimately excluded from the meta-analysis.

### 2.5 Data collection and data items

In this systematic review, data from each of the included studies were extracted, encompassing the first author’s name, publication year, study design, study population, sample size, country, mean age, sex, ILD diagnosis criteria, air pollution metrics, and the key findings. We collected risk estimates from the preferred model of choice by each study author (some of which were presented in abstracts). These estimates included hazard ratios (HR), risk ratios (RR), or odds ratios (OR), along with their corresponding 95% confidence intervals (CIs). We then entered the extracted data from the articles into an Excel spreadsheet for analysis. To ensure reliability, the primary author (DL) extracted the complete data, while co-author (CF) independently verified the accuracy and completeness of the extracted information. Any disagreements were adjudicated and resolved by senior investigator (TC).

### 2.6 Study risk of bias assessment

The risk of bias (30) of included studies was performed using the Newcastle-Ottawa Scale (NOS), a validated tool for assessing the quality of nonrandomized studies (31). It follows a “star system” approach, evaluating three perspectives: selection of study groups, comparability of groups, and ascertainment of exposure or outcome of interest in case–control or cohort studies. Each item is assigned one point, except for comparability, which can score up to two points.

To ensure precision and clarity in evaluating the comparability section of this review, a study receives one star if it controls or adjusts the age and sex for analysis. Additionally, if the study incorporates additional factors, such as smoking status, forced vital capacity (FVC), antifibrotic treatment, average temperature, and humidity, into the analysis to adjust for potential confounding effects, it qualifies for an additional star.

The adequacy of follow-up time of the cohorts was determined based on the assessed outcomes within each study, including long-term and short-term outcomes according to the definition provided in the 2021 World Health Organization’s (WHO) Air Quality Guidelines (AQG). Long-term exposure is characterized by an average measurement taken over one or several years, while short-term exposure is defined as a measurement taken over a period ranging from minutes to days (26).

The maximum possible score is nine. Quality is categorised as good, fair, or poor based on stars awarded in each domain. The majority of the included studies in this review were cohort and case–control studies. The evaluation of bias risk in these types of studies using the NOS was conducted independently by two investigators (DL and CF). It is worth noting that for ecologic studies, there is no established and validated tool for evaluation of quality (32). As a result, the assessment of the five ecologic studies was primarily descriptive. The remaining one study is a panel study. Its limitations are also described.

### 2.7 Data analysis

Due to the scarcity of studies focusing on the same pollutant and health outcome, the findings are presented through a narrative synthesis method. Meta-analyses were performed exclusively when there was a minimum of three studies examining identical pollutant-health outcome pairs, all evaluating equivalent increments in pollutant concentration. Due to variations in study design, methodology, and study populations, a random effects model was implemented.

For meta-analysis, given the variations in methodology across the studies, an inverse-variance approach was applied for the assessment of their relative significance (33). Within each study, we computed the standard error (SE) and the natural logarithm (log) of both Hazard Ratios (HR) and Odds Ratios (OR). These log-transformed HR and OR values were treated as equivalent to log Risk Ratio (logRRs). Alongside the SE, we calculated the pooled Risk Ratio (RR) for the meta-analysis. This approach mirrors the methodology previously employed (34). The 0.05 level (two-sided) was used to indicate significance of *p*-values.

For studies included in the meta-analysis, the RRs were used for the measure of association between air pollutants and health outcomes for comparing equivalent increases in pollutant concentration. In cases where RRs or ORs for gaseous pollutants were in parts per billion (ppb), they were converted to μg/m^3^ at standard conditions (25°C) first. The heterogeneity in effect sizes was assessed using the I^2^ statistic (35). The consideration of publication bias was deemed unnecessary due to the limited number of studies addressing the same pollutant and health outcome. All statistical analyses and meta-analyses were conducted with RevMan 5.4 software (Cochrane Collaboration).

## 3 Results

### 3.1 Study selection

**Figure 1:**
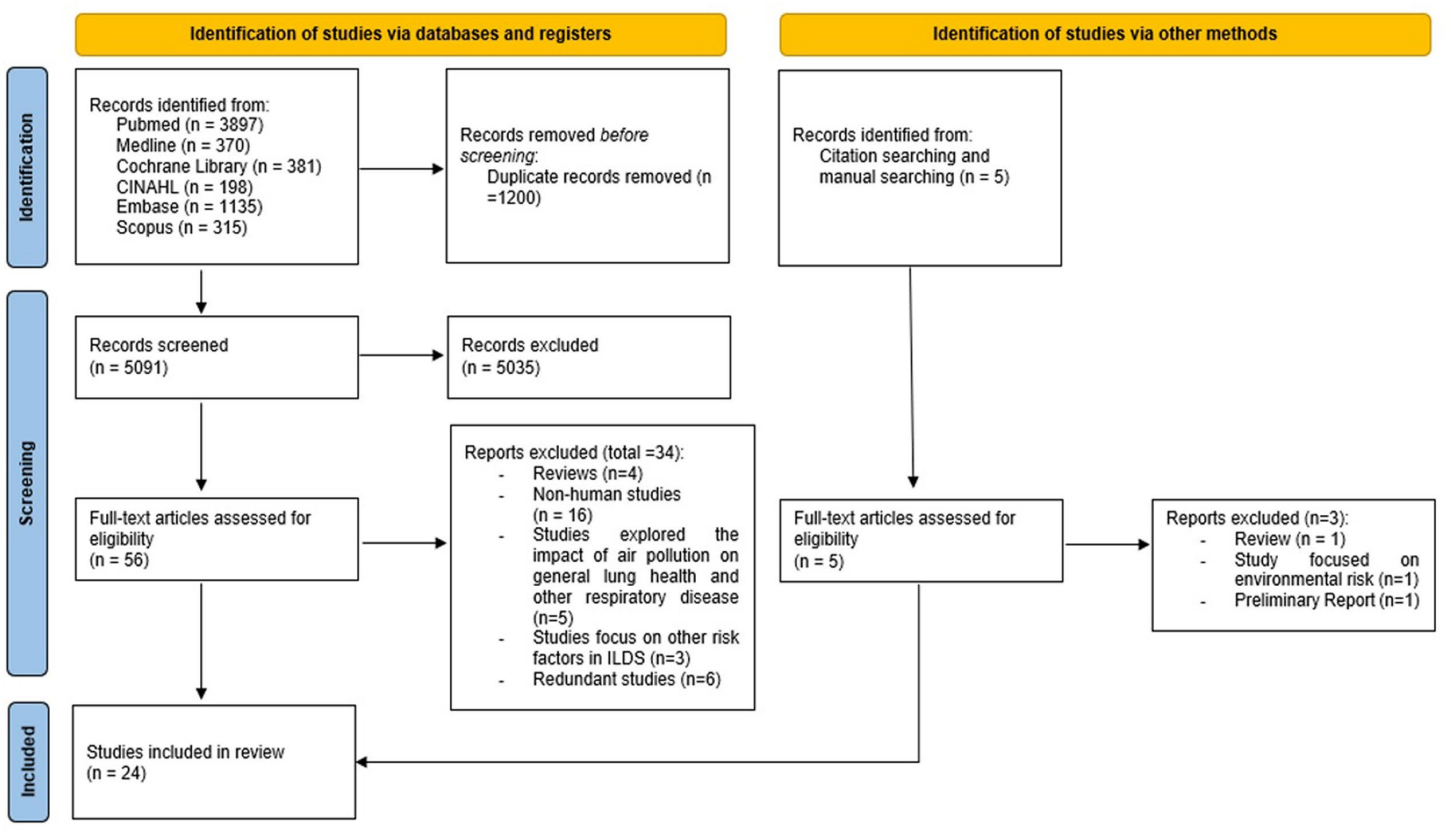
PRISMA 2020 flow diagram for study selection.

The study selection process is summarised in Figure 1. Initially, a total of 6,291 records were identified through database searching. All search results from the database were imported into Endnote 20 to remove duplicates. After eliminating 1,200 duplicates, 5,091 records remained and underwent the initial screening based on their titles and abstracts. Subsequently, 5,035 records were excluded based on specific inclusion and exclusion criteria. The remaining 56 studies underwent a comprehensive review. During this review, 34 records were deemed ineligible and were subsequently removed, leaving a total of 22 papers sourced from the electronic database for inclusion in this literature review. Among the 34 ineligible papers, six contained duplicate content that was already present in other included papers, despite having different titles. Therefore, these six duplicate papers were removed from the selection to avoid redundancy in the literature review (36–41).

Five additional records were identified through citation and manual searching. One study emerged as a preliminary report (42). An attempt to communicate with the authors to gain a deeper comprehension of methods and analysis was unsuccessful, resulting in the exclusion of that report. Of the remaining four studies, two meeting the eligibility criteria were included. In summary, our systematic literature review comprised a total 24 papers (Figure 1).

### 3.2 Study characteristics

**Table 2:** Summary of studies examined the impact of air pollution on interstitial lung disease (ILDs).

First author & year (reference)	Study population	Sample size	Country	Age years (mean ± SD)	Males/females	ILD diagnosis criteria	Air pollution metrics	Key findings
Johannson K. A., 2014 (43)	Patients with IPF	436	South Korea	62.8 ± 7.9	345/91	IPF diagnosis re-confirmed as per 2011 ATS/ERS/JRS/LARA Guideline	Mean and maximum Levels of O_3_, NO_2_, PM _10_, SO_2_ and CO over the 6 weeks preceding the date of AE. Cumulative exposures were calculated over the entire period, from IPF diagnosis to date of censoring.	Higher exposure to O_3_ and NO_2_ in the preceding 6 weeks increased the risk of acute exacerbation in IPF. Air pollution potentially contributes to the development of acute exacerbation in IPF. No statistically significant correlation was found between cumulative exposure to air pollution and mortality.
Sack C., 2017 (44)	Participants of the Multi-Ethnic Study on Atherosclerosis (MESA)	2,265	United States	59.8 (9.4)	1059/1206	ILD defined based on HRCT scans	Annual average exposure levels of PM _2.5_, NO_X_, NO_2_, and O_3_	The odds of Interstitial lung abnormalities (ILAs) increased 1.77-fold per 40 ppb increment in NOx (95% CI 1.06 to 2.95, *p* = 0.03). No association was found between ILAs and ambient PM_2.5_, O_2_, or O_3_ concentrations.
Conti S., 2018 (45)	Inhabitants of Lombardy, Italy	Almost 10 million inhabitants	Italy			IPF diagnosis based on International Classification of Diseases (ICD-9-CM) code 516.3	Average concentrations over 2005–2010 for NO_2_ and O_3_, and over 2005–2009 for PM_10_	The incidence of IPF was not associated with the concentration of PM_10_ and O_3_. There is a significant association between the incidence of IPF and cold season NO_2_ concentration: a 10 μg·m − 3 increase in NO_2_ is associated with a 7.93% (95% CI 0.36–16.08%) higher incidence rate of IPF. Inhalation of traffic-related pollutants, such as NO_2_, may be involved in the development of IPF.
Johannson K. A., 2018 (46)	Patients with IPF	25	United States	73.6 ± 7.5	21/4	IPF diagnosis re-confirmed as per 2011 ATS/ERS/JRS/LARA Guideline	Mean levels of O_3_, NO_2_, PM_2.5_, and PM_10_ over 2 to 5 weeks preceding clinical measurements; mean and maximum level over 40 weeks period	Higher average exposures to NO_2_, PM_2.5_, and PM_10_ were associated with lower FVC in patient with IPF. Week-to-week changes in FVC were independent of air pollution exposure.
Sesé L., 2018 (47)	Patients with IPF	192	France	67.9 ± 11.2	148/44	IPF diagnosis based on 2000 ATS/ERS diagnosis criteria	Short-term effect: mean level of O_3_, NO_2_, PM_2.5_, and PM_10_ over the 6 weeks preceding the date of AE. Long-term effect: cumulative exposure levels from IPF diagnosis to the date of the event or censoring	Onset of acute exacerbation (AE) was significantly associated with an increased mean level of ozone in the six preceding weeks. Mortality was significantly associated with increased levels of long-term exposure to PM_10_ and PM_2.5_. No association was observed between AE or Mortality and NO_2_. Disease progression was not associated with increased cumulative concentrations of NO_2_, O_3_, PM_10_ or PM_2.5_
Winterbottom C. J., 2018 (48)	Patients with IPF	135	United States	68 (46–92)	101/34	IPF diagnosis re-confirmed as per 2011 ATS/ERS/JRS/LARA Guideline	Daily mean levels of PM 2.5 and PM_10_ between 2007 and 2013	Increased exposure to PM_10_ was significantly associated with accelerated FVC decline, with each 5 mg/m^3^ increase corresponding to a 35 cc/y decline in FVC (95% CI, 6–65 cc/y). Increased exposure to PM_2.5_ was significantly related to an accelerated rate of oxygen use increase during the 6MWT, with each 5 μg/m^3^ increase corresponding to a 1.15 L/y increase (95% CI, 0.03–2.26 L/y). No association was found between the proximity of subjects’ residences to major roads and highways.
Pirozzi C., 2018 (49)	Patients with fibrotic sarcoidosis	16	United States	59 (53.25, 62.5)	4/12	Sarcoidosis diagnosis based on American Thoracic Society (ATS) criteria (1999)	Average Levels of PM_2.5_ and O_3_ for 7, 10, and 14 days preceding each study visit	Short-term PM_2.5_ exposure was linked to increased severity of respiratory and QoL symptoms according to the KSQ. PM_2.5_ exposure was not associated with FEV1, FVC, episodes of FEV1 decline >10%, or respiratory symptoms measured by SGRQ or LCQ. Short-term ozone exposure was not associated with any health outcomes measured by the SGRQ, LCQ, or KSQ.
Rice M. B., 2019 (50)	Participants from Framingham study	2,618	United States	59.5 ± 11.9	1299/1319	ILAs diagnosis Based on CT scans	5-year (2004–2008) average levels of PM_2.5_, Elemental carbon (EC) and O_3_	Higher long-term exposure to EC was associated with increased odds and progression of ILAs on chest imaging. No significant association was found between PM_2.5_ or O_3_ and the incidents or progression of ILAs.
Dales R., 2020 (51)	Patients with a primary diagnosis of IPF	3,989	Chile		1665/2324	IPF diagnosis based on International Classification of Diseases, 10th Revision (ICD-10) J84.1	Annual average of 24 h means of CO, NO_2_, SO_2_, PM_2.5_ and PM _10_, and 8-h daily maximum level of O_3_ over 0 to 6 days before admission and over the 30 days before admission	NO_2_ and PM_10_ are the strongest independent pollutants associated with increased hospitalization. CO and SO_2_ had significant positive association with hospitalisation (*p* < 0.05), but lost association when use two pollutant models. The air pollution levels within 3 days before admission had the most substantial impact on IPF patients. Acute increases in air pollution are a risk factor for hospitalization of patients with a primary diagnosis of IPF.
Chen H. H., 2020 (52)	Patients with connective tissue diseases (CTDs)-ILD	505 CTD-ILD and 2020 non-ILD CTD	Taiwan, China	CTD-ILD 60.1 ± 14.7/ non-ILD CTD 59.4 ± 14.0	CTD-ILD 125/380 non-ILD CTD 500/1,520	CTD-ILD diagnosis based on ICD-9 code 515 and 516.36	Mean levels of PM_2.5_, PM_10_, NO_2_, CO, SO_2_ and O_3_ 1 year prior to the index date.	Patients with CTD-ILD had slightly lower average exposure to PM_2.5_, PM_10_, and SO_2_ compared to patients with non-ILD CTD, but not statistically significant. O_3_ exposure (10 ppb) (aOR, 0.51; 95%CI, 0.33 to 0.79) showed an inverse association with the risk of ILD development in patients with CTD in Taiwan.
Mariscal-Aguilar P., 2021 (53)	Patients with IPF	52	Spain	66 ± 10	41/11	IPF diagnosis based on ATS/ERS criteria (unsure which version)	Mean levels of PM_2.5_, PM_10_, NO_2_, CO, SO_2_, and O_3_ (from the previous 12 weeks prior to death for mortality, mean level between 2013 and 2019 for disease progression.)	Increased exposure to carbon monoxide (CO) was significantly associated with increased mortality. No association was found between any of the other investigated air pollutants and IPF mortality or severity.
Shull J. G., 2021 (54)	Patients with IPF	379	Spain			IPF diagnosis based on central expert ILD multidisciplinary discussion (MDD)	Annual mean concentration of PM_2.5_ over 1 year in 2015	Prevalence of IPF is higher in areas of elevated PM_2.5_ concentration. Mapping IPF can help identify geographic regions at higher risk for disease development and potential triggers.
Tahara M., 2021 (55)	Patients with idiopathic interstitial pneumonias (IIPs) (152 IPF and 200 other subtypes of IIPs)	352	Japan			IIPs diagnosis based on HRCT, surgical lung biopsy (SLB) samples and MDD	Daily and monthly mean concentrations of SO_2_, NO, NO _2_, NOx, CO, O _3,_ PM _2.5_ and PM _10_	Short-term exposure to PM_2.5_ is associated with a significant risk of acute exacerbation (AE) of IPF. Each 10 μg/m^3^ increase in PM_2.5_ amplified the risk of AE-IPF by approximately 2.5 times. Significant positive associations were observed between the monthly mean exposure to NO, NO_2_, NOx, PM _2.5_, and AE-IIPs. No significant relationship was observed between AE-IPF incidence and exposure to SO_2_ and O_3_.
Tomos I., 2021 (56)	Patients with IPF	118	Greece	72 ± 8.3	88/30	IPF diagnosis based on 2011 ATS/ERS/JRS/LARA Guideline	Annual mean levels of O_3_, NO_2_, PM_10_ and PM_2.5_	Long-term exposure to NO_2_, PM_2.5_, and PM_10_ increased the risk of AE-IPF, independent of age, lung function impairment, anti-fibrotic treatment, and smoking status. Long-term exposure to O_3_ is positively linked to increased serum IL-4 levels (an inflammatory mediator) (*p* = 0.014), whilst PM_2.5_, PM_10_ and NO_2_ were inversely associated with %changes of IL-4 (p = 0.003, *p* = 0.003, *p* = 0.032) and osteopontin (p = 0.013, *p* = 0.013, *p* = 0.085) in AE-IPF patients.
Yoon H-Y, 2021 (57)	Patients with IPF	1,114	South Korea	65.7 ± 8.2	897/217	IPF diagnosis based on 2011 ATS/ERS/JRS/LARA Guideline and MDD	Annual mean levels of PM_10_ and NO_2_	Exposure to NO_2_ is associated with increased mortality risk in patients with IPF, especially in elderly males. Each 10-ppb increase in NO_2_ concentration is linked to a 17% higher mortality rate. PM_10_ was not associated with IPF mortality in all patients and in subgroups stratified by age or sex.
Goobie G. C., 2022 (58)	3 cohort patients with fibrotic interstitial lung disease (fILD)	6,683	United States and Canada	67 (57–73)	3653/3030	fILD diagnosis were made by specialist ILD physicians: IPF diagnosis as per ATS/ERS/JRS/ALAT 2018; Hypersensitivity pneumonitis diagnosis as per ATS/JRS/ALAT 2020	Monthly mean exposures 5 years pre-enrolment or precensoring for total PM _2.5_ and its constituents (SO_4_^2−^, NO_3_^−^, NH_4_^+^, black carbon, organic matter, sea salt, and soil)	PM_2.5_ exposure of 8 μg/m^3^ or higher was associated with higher mortality risk across all cohorts. Increasing exposure to sulfate, nitrate, and ammonium PM_2.5_ constituents was associated with increased mortality, lower baseline lung function, and increase in Lung Function decline, and more rapid disease progression in patients with fILD.
Liang L., 2022 (59)	Patients with IPF	11,974 IPF admissions	China			Primary discharge diagnosis of IPF based on ICD-10: J84.1	Daily mean concentrations of PM_10_, PM_2.5_, NO_2_, O_3_, SO_2_	Significant association between higher PM_2.5_ concentrations and increased risk of IPF hospitalization at no time lag(lag0) and short-term exposure periods. Significant positive associations were observed at lag0 between IPF hospitalization and SO_2_, O_3_, and NO_2_ in men only. Positive associations were seen for moving averages of 0–30 days between IPF hospitalization and PM_10_, NO_2_, and SO_2_, but not with PM_2.5_ or Ozone.
Liu B., 2022 (60)	Patients hospitalized for rheumatoid arthritis associated with interstitial lung disease (RA ILD)	221 RA-ILD admissions	China			Diagnosis of RA-ILD as per 1987 ACR criteria for RA, ILD by HRCT reviewed by rheumatologist and radiologist.	Monthly mean concentrations of PM_2.5_, PM_10_, SO_2_, O_3_ and NO_2_	There was a significant correlation between ambient air pollutant concentrations (PM_2.5_, PM_10_, SO_2_, and NO_2_) and monthly hospitalizations for RA-ILD. PM_2.5_, PM_10_, and SO_2_ had the most significant effect on the month (lag 0), and NO_2_ was most related to RA-ILD at a lag of two months (lag 2). For each 10μg/m^3^ increase in PM_2.5_, PM_10_, SO_2_ and NO_2_, the monthly admissions of RA-ILD increased by 0.875, 0.548, 1.968, and 1.534%, respectively. Higher O_3_ levels to be inversely associated with hospitalizations for RA-ILD.
Cui F., 2023 (61)	Participants from the UK Biobank	433,738	United Kingdom	56.4 ± 8.1	199,363/ 234,375	IPF diagnosis based on ICD-10: J84.1	Annual average concentrations of NO_2_, NOx, PM_2.5_ and PM 10	The mean (median) concentrations of NO_2_, NOx, PM_2.5_ and PM_10_ were 26.65 (26.13), 43.99 (42.21), 9.99 (9.93) and 16.23 (16.03) μg·m^−3^ Significantly increased risk of incident IPF was associated with individual exposures to NO_2_, NOx and PM_2.5_. The combination of air pollutants and genetic susceptibility has additive effects on IPF risk. Participants with high polygenic risk scores (PRS) and high air pollution had the highest risk of incident IPF.
Goobie G. C., 2023 (62)	Two cohort of IPF patients	1,059	United States	69 (61–74)	772/287	IPF diagnosis made by a specialist ILD clinician, as per clinical practice guidelines	Monthly mean of PM_2.5_ (from 2000 to 2018) and its constituents (SO_4_^2−^, NO_3_^−^, NH_4_^+^, black carbon, organic matter, sea salt, and soil) (from 2000 to 2017)	Higher PM_2.5_ and its constituent exposures were associated with higher global DNA methylation (DNAm) in patients with IPF. Global DNAm may serve as a biomarker of short and long-term exposures to ambient pollution in IPF patients.
Mariscal-Aguilar P., 2023 (63)	Patients with IPF	69	Spain	73.70 ± 7.72	53/16	IPF diagnosis based on patients’ medical record at the time of each admission	Mean concentration of CO, NO_2_, O_3_, and NOx over 1, 3,6, 12 and 36 months before an event	Higher average values of CO, NO_2_, and NOx were significantly associated with an increased likelihood of chronic respiratory failure in different periods in IPF patients. Significant associations were found between the average levels of NO_2_, O_3_, and NOx and the probability of hospital admissions due to respiratory causes and mortality in these patients.
Yoon H. Y., 2023 (64)	Patients with IPF	946	South Korea	65.4 ± 8.1	765/181	IPF diagnosis based on 2011 ATS/ERS/JRS/LARA Guideline and MDD	Annual average concentrations of NO_2_ and PM_10_	Exposure to NO_2_ increases the risk of disease progression in IPF. A 10-ppb increase in NO_2_ concentration was associated with a 10.5% increased risk of progression. No association was found between PM_10_ and disease progression in IPF patients.
Roeser A., 2023 (65)	Patients with Systemic sclerosis (SSc)-associated ILD	181	France	53 (42.5–64)	37/144	SSc diagnosis based on 2013 American College of Rheumatology/European League Against Rheumatism (ACR/EULAR) classification criteria and ILD diagnosis based on 2018 ATS/ERS/JRS/ALAT Guidelines	Annual mean concentrations of PM_2.5_, PM_10_, NO_2_, and O_3_	The mean (SD) exposure levels during the 5 years preceding ILD diagnosis for the study population were 27.9 (8.4) μg/m^3^ for NO_2_ (range 9.9–42.2), 44.2 (6.0) μg/m^3^ for O_3_ (range 36.5–74.6), 23.5 (3.3) μg/m^3^ for PM_10_ (range 15.6–30.0) and 15.6 (2.4) μg/ m^3^ for PM_2.5_ (range 9.6–20.3). High levels of O_3_ exposure are associated with more severe SSc-associated ILD at diagnosis, and progression at 24 months. No significant associations were found for PM exposure or NO_2_ exposure
Zheng Q., 2023 (66)	Patients with IPF	570	Australia	70.9 (8.2)	401/169	IPF patients from Australian IPF Registry – IPF diagnosis made by referring respiratory physician	5-year annual mean concentrations of PM_2.5_ and NO_2_	Living near a major road and increased PM_2.5_ were both associated with an increased rate of annual decline in DLco, but not associated with a faster decline in FVC. No association was found between NO_2_ and lung function decline. Median (25th–75th percentiles) annual PM_2.5_ and NO_2_ were 6.8 (5.7, 7.9) μg/m^3^ and 6.7 (4.9, 8.2) ppb. No significant association of increased PM_2.5_ (HR 1.16, 95% CI 0.88 to 1.53) or NO_2_ (HR 1.06, 95% CI 0.85 to 1.33) with rapid progression of IPF.

O_3_, ozone; NO_2_, nitrogen dioxide; PM, particulate matter; PM_2.5_, particulate matter with a diameter of 2.5 micrometres or less; PM_10_, particulate matter with a diameter of 10 micrometres or less; SO_2_, sulfur dioxide; CO, carbon monoxide; NO, Nitric Oxide; IPF, idiopathic pulmonary fibrosis; ILAs, interstitial lung abnormalities; FVC, Forced vital capacity; AE, Acute exacerbation; NOx, Nitrogen Oxides; SGRQ, Saint George Respiratory Questionnaire (SGRQ) measures the impact of respiratory symptoms on overall health, daily life, and perceived well-being, and queries symptoms over the past three months and “these days”; LCQ, Leicester cough questionnaire (LCQ) is a 19-item quality of life (QoL) measure of cough over the prior two weeks; KSQ, King’s Sarcoidosis Questionnaire (KSQ) is a validated questionnaire for assessing general health status and lung specific health status; Elemental carbon (EC), a traffic related PM_2.5_ constituent; DLco, diffusing capacity of the lungs for carbon monoxide; SO_4_^2−^, sulfate; NH_4_^+^, ammonium; NO_3_^−^, nitrate; PFF, Pulmonary Fibrosis Foundation; Simmons, Simmons Center for Interstitial Lung Disease Registry; aOR, adjusted odds ratio; aHR, adjusted hazard ratio.

Studies were conducted across various countries, representing diversity of geography, culture, and population ethnicity (Table 2). Six studies were conducted in the United States (44, 46, 48–50, 62), representing a quarter of all the included studies. Three studies were conducted each in Spain (53, 54, 63) and South Korea (43, 57, 64). Two studies each came from France (47, 65) and mainland China (59, 60). The remaining studies were conducted in Italy (45), Chile (51), Taiwan (52), Japan (55), Greece (56), the United Kingdom (61), and Australia (66). A collaborative effort between investigators from the USA and Canada resulted in a study evaluating North American ILD populations and air pollution (58).

All 24 selected studies were observational in nature. Among these, 17 studies were published in the last 5 years (2019–2023), while the remaining seven studies were published between 2014 and 2018 (43–49). Population sizes varied widely, ranging from 16 participants to 10 million inhabitants. The mean population ages ranged from 53 to 73.7 years old. Of the studies that reported sex distribution, 207,108 (46.5%) participants were male, and 238,166 (53.5%) participants were female.

The majority of studies (20 studies) enrolled participants from individuals diagnosed with ILD groups, with the exception of four studies that recruited individuals from the general community (44, 45, 50, 61). Among the patient groups with ILD participants, 14 studies included participants with IPF only in their study populations. Five studies focused on non-IPF ILD patients including fibrotic sarcoidosis (49), a connective tissue disease (CTD) population with and without associated ILD (52), fibrotic ILD (fILD) (58), rheumatoid arthritis associated ILD (RA-ILD) (60), and systemic sclerosis (SSc)-associated ILD (SSc-ILD) (65). The last study is a case–control study that involved individuals who had received diagnoses of idiopathic interstitial pneumonias (IIPs) (55) including those with IPF.

Among the 24 studies incorporated in the analysis, seven studies scrutinized the link between short-term exposure to air pollution and its impact on health outcomes (43, 46, 49, 51, 55, 59, 60). Fourteen studies delved into the connection between long-term exposure to air pollution and health outcomes (44, 45, 48, 50, 52, 54, 56–58, 62, 64–66). The remaining three studies examined the effects of both short-term and long-term exposure (47, 53, 63).

### 3.3 Risk of bias in studies

Eighteen of the 24 analysed studies were cohort or case control studies, and their assessment was conducted using the NOS tool as described above. NOS scores were assigned to each study by consensus. Among the appraised 18 studies, 12 received a full score of 9/9 (43, 44, 47, 48, 50, 56–58, 61, 62, 65, 66), indicating high quality research. Four studies received a score of 8/9 (49, 52, 53, 64), one study received a score of 7/9 (55), and one study was scored as 6/9 (46). All 18 studies were considered to be of good to high quality.

The limitations identified in the remaining six studies involved data sources, accuracy of diagnoses, measurement of exposure, and potential confounding factors. Most studies had limitations of potential underestimation or misclassification of cases due to data sources or diagnostic coding (45, 51, 54, 59). Additionally, there was a lack of adjustment for individual-level factors (45, 51, 59, 60), and limited data on certain pollutants (54, 59) or occupational history (45, 59). Some studies had limited statistical power due to small sample size and limiting generalisability to broader populations or regions (60, 63) (Table 3).

**Table 3:** Critical appraisal of selected studies.

				Newcastle-Ottawa scale appraisal				
First author & year (reference)	Study design	Sample size	Limitations	Selection	Comparability	Outcome	Score	Quality
Johannson, 2014 (43)	Cohort	436		****	**	***	9	Good
Sack C., 2017 (44)	Cohort	2,265		****	**	***	9	Good
Johannson, 2018 (46)	Cohort	25		***	**	*	6	Good
Sesé, 2018 (47)	Cohort	192		****	**	***	9	Good
Winterbottom, 2018 (48)	Cohort	135		****	**	***	9	Good
Pirozzi, 2018 (49)	Cohort	16		***	**	***	8	Good
Rice, 2019 (50)	Cohort	2,618		****	**	***	9	Good
Mariscal-Aguilar, 2021 (53)	Cohort	52		***	**	***	8	Good
Tomos, 2021 (56)	Cohort	118		****	**	***	9	Good
Yoon, 2021 (57)	Cohort	1,114		****	**	***	9	Good
Goobie, 2022 (58)	Cohort	6,683		****	**	***	9	Good
Cui, 2023 (61)	Cohort	433,738		****	**	***	9	Good
Goobie, 2023 (62)	Cohort	1,059		****	**	***	9	Good
Yoon, 2023 (64)	Cohort	946		****	**	**	8	Good
Roeser, 2023 (65)	Cohort	181		****	**	***	9	Good
Zheng, 2023 (66)	Cohort	570		****	**	***	9	Good
Chen, 2020 (52)	Case–Control	2,525		****	**	**	8	Good
Tahara, 2021 (55)	Case–Control	352		***	**	**	7	Good
Conti, 2018 (45)	Ecologic	Almost 10 million inhabitants	1. Potential underestimation of incident cases of IPF due to reliance on administrative databases. 2. Uncertainty regarding the accuracy of diagnosis codes. 3. Proxy use of incident diagnosis date as disease onset. 4. Limited data on individuals who moved before 2005. 5. Potential residual confounding from unmeasured risk factors such as occupation and smoking.					
Shull, 2021 (54)	Ecologic	379	1. Inability to quantify precise air pollution exposure prior to diagnosis. 2. Potential underestimation of prevalence in rural areas and small towns. 3. Other pollutants such as NO_2_ and O_3_ were not examined.					
Liang, 2022 (59)	Ecologic	11,974	1. Ecological study design limits individual-level predictions. 2. Individual-level factors like smoking and socioeconomic status were not adjusted for. 3. Lack of data on time-varying factorsand specific residential addresses. 4. Outcome measurement limitations due to the primary diagnosis of IPF was coded by clinicians at discharge, which may have led to misclassification. 5. Constraints in analysing multiple pollutants and lack of occupational history.					
Liu, 2022 (60)	Ecologic	221	1. Exclusion of hospital outpatients may underestimate the impact of exposure. 2. Use of average daily data from fixed-site monitoring stations may not reflect individual exposure levels accurately. 3. Low statistical power due to limited incidences of RA-ILD. 4. Study conducted in a single district, limiting generalizability of results					
Dales, 2020 (51)	Ecologic	3,989	1. Lack of detailed personal information relevant to interstitial disease like smoking. 2. Lack of a more detailed description of the diagnosis. Inaccuracies in disease definition due to errors in both clinical diagnosis and diagnostic coding.					
Mariscal-Aguilar, 2023 (63)	Panel	69	1. Two-centre and limited sample size. 2. Lack of consideration for workplace air pollution. 3. Missing data on temperature and humidity. 4. Inability to control for all confounding factors. 5. Lack of diversity in exposure levels since the majority of patients reside in the same region.					

Thresholds for converting the Newcastle-Ottawa scales to AHRQ standards (good, fair, and poor): good quality: 3 or 4 stars in selection domain AND 1 or 2 stars in comparability domain AND 2 or 3 stars in outcome/exposure domain. Fair quality: 2 stars in selection domain AND 1 or 2 stars in comparability domain AND 2 or 3 stars in outcome/exposure domain. Poor quality: 0 or 1 star in selection domain OR 0 stars in comparability domain OR 0 or 1 stars in outcome/exposure domain.

### 3.4 Findings: impact of air pollutants on ILDs

Air pollution have been found to have multiple impacts on ILDs (Supplementary Table S1).

#### 3.4.1 Air pollution and mortality in IPF or fibrotic interstitial lung disease (fILD)

Six studies involving 8,546 participants examined the association between air pollution and mortality in individuals with IPF (43, 47, 53, 57, 63) or fILD (58). Among these studies, five studies investigated NO_2_ (43, 47, 53, 57, 63), four studies involved O_3_ (43, 47, 53, 63), and three studies each examined PM_10_ (47, 53, 57), and PM_2.5_ (47, 53, 62).

The findings regarding the association between NO_2_ and O_3_ were inconclusive. Specifically, three studies did not detect any association between the average levels of NO_2_ and O_3_ in the 12 weeks preceding an individual’s death (53), the cumulative long-term NO_2_ and O_3_ exposure from the time of IPF diagnosis to the event date (43, 47), and IPF mortality. However, Mariscal-Aguilar et al. (63) reported a link between elevated mean levels of NO_2_ and O_3_ and higher probability of IPF mortality in individuals exposed to these pollutants for 1–3 months for O_3_, and 2–36 months for NO_2_. Additionally, Yoon et al. (57) found a positive association between NO_2_ and IPF mortality (95% CI 1.030–1.344, *p* = 0.016).

Variable impact of PM_10_ exposure has been described. Although two studies (53, 57) failed to detect any connection between short and long-term mean levels of PM_10_ and IPF mortality, a separate study (47) revealed a positive link. This study demonstrated that long-term cumulative exposure to elevated PM_10_ levels was associated with increased IPF mortality (HR 2.01; 95% CI 1.07–3.77; *p* = 0.03).

Regarding PM_2.5_ exposure, Mariscal-Aguilar et al. (53) reported no discernible link between the mean level of PM_2.5_ over the 12 weeks preceding death and IPF mortality, whereas Sesé et al. (47) revealed a significant correlation between long term cumulative exposure and IPF mortality (HR 7.93; 95% CI 2.93–21.33; *p* < 0.001). Another study exploring the effects of PM_2.5_ and its constituents on mortality in patients with fILD (58) showed that PM_2.5_ exposure of 8 μg/m^3^ or higher over a 5-year period was associated with increased mortality across all fILD patient groups (HR, 1.18; 95% CI, 1.02–1.37; *p* = 0.03).

In Mariscal-Aguilar et al. study (53), the mean CO level assessed 12 weeks before death was associated with increased IPF mortality (OR 2.45; 95% CI 1.39–4.56; *p* = 0.005). Conversely, Mariscal-Aguilar et al. (63), did not find any association between CO exposure and IPF mortality. However, this same study did reveal a positive association between increased mean concentrations of nitrogen oxides (NOx) and IPF mortality over a 12-36-month period (63).

#### 3.4.2 Air pollution and disease progression (IPF and systemic sclerosis-associated ILD, SSc-ILD)

Three studies (1708 participants) explored the link between air pollution and the progression of IPF (47, 64, 66). Among the four pollutants, namely PM_10_, PM_2.5_, O_3_, and NO_2_, the results showed that PM_10_ (47, 64), PM_2.5_ (47, 66), and O_3_ (47) had no relationship with the disease progression in IPF patients. However, the results regarding NO_2_ were inconclusive.

In the study conducted by Sesé et al. (47), researchers examined cumulative concentrations of these pollutants from the date of IPF diagnosis to the event, while Zheng et al. (66) assessed 5-year annual mean concentrations. Neither study found a link between NO_2_ levels and progression of IPF. However, Yoon et al. study (64) reported that a 10-ppb increase in NO_2_ concentration was associated with a 10.5% increase in the risk of IPF disease progression (HR 1.105; 95% CI 1.000–1.219, *p* = 0.048) after adjusting for covariates.

Additionally, a study of 181 participants with SSc-ILD, examined the relationship between air pollution and the severity at diagnosis and with progression of ILD at 24 months (65). Increased O_3_ mean (SD) exposure levels during the 5 years preceding ILD diagnosis were associated with worse disease severity at diagnosis (adjusted OR: 1.12, 95% CI 1.05–1.21; *p* = 0.002) and disease progression at 24 months (OR: 1.10, 95% CI 1.02–1.19; *p* = 0.02), while PM_2.5_, PM_10_, and NO_2_ showed no association with the severity or progression of ILD.

Furthermore, one study involving 181 participants diagnosed with SSc-ILD investigated the association between air pollution and the severity of the disease at diagnosis and its progression over a 24-month period (65). Elevated levels of O_3_ exposure were significantly linked to more severe SSc-ILD both at the time of diagnosis (aOR: 1.12, 95% CI 1.05–1.21; *p* = 0.002) and progression at 24 months (aOR: 1.10, 95% CI 1.02–1.19; *p* = 0.02). However, no significant associations were identified between exposure to particulate matters (PM_10_ and PM_2.5_) or NO_2_ and the disease severity or its progression.

#### 3.4.3 Air pollution and the risk of acute exacerbation of ILD (AE-IPF and AE-IIP)

Four studies (1,098 participants) investigated the potential association between air pollution and acute exacerbation of IPF (AE-IPF) (43, 47, 55, 56) including O_3_, NO_2_, PM_10_, PM_2.5_, SO_2_, CO, NO and NOx. Among these, SO_2_ and CO were examined in two of the studies, with no association found between short-term exposure to higher levels of these pollutants and incidence of AE-IPF (43, 55). A study conducted by Tahara et al. (55) also examined NO and NOx, with significant and near-significant association between increase in NO (OR 1.46; 95% CI 1.11–1.93; *p* = 0.008) and NOx (OR 1.24, 95% CI 0.99–1.53; *p* = 0.052) concentrations and AE-IPF.

**Figure 2:**
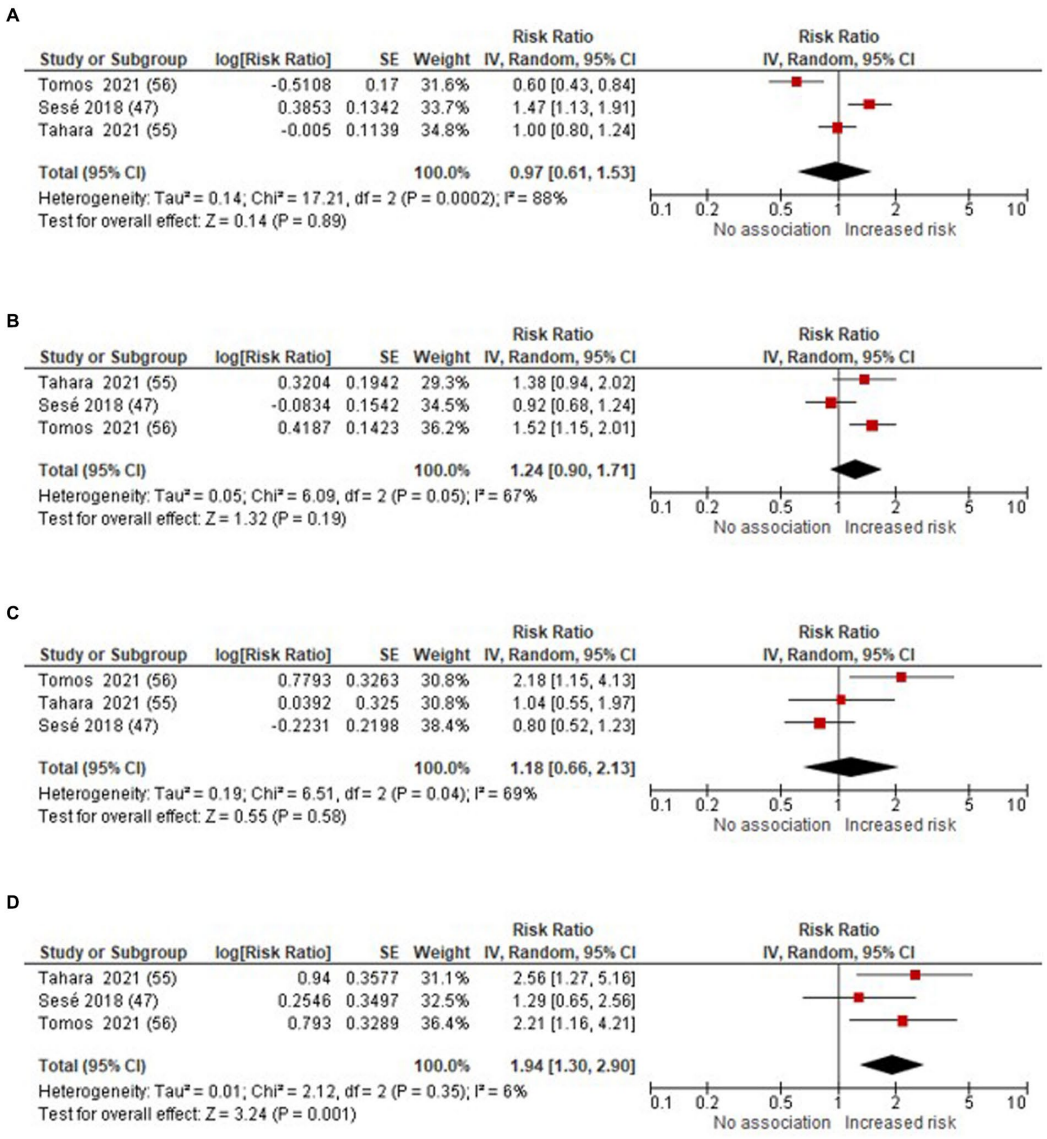
Forest plot: air pollutants (per 10 μg/m^3^ increase in air pollutants concentrations) and the risk of AE-IPF **(A)** O_3_ **(B)** NO_2_ **(C)** PM_10_ **(D)** PM_2.5_.

We conducted meta-analyses to assess the RRs for AE-IPF in relation to exposure to every 10 μg/m^3^ increment in air pollutant concentrations, including O_3_, NO_2_, PM_10_, and PM_2.5_ (as illustrated in Figure 2). The study by Johannson et al. (43) expressed effect sizes per standard deviation increase in pollutant. However, it was excluded because we could not locate, in the main or supplementary text, a description of the SD on a continuous scale (43).

The meta-analysis revealed a significant association between the increased risk of AE-IPF in PM_2.5_, yielding RR 1.94 (95% CI 1.30–2.90; *p* = 0.001). However, the link between O_3,_ NO_2_ and PM_10_ and the risk of AE-IPF remained uncertain, with RR 0.97 (95% CI: 0.61–1.53; *p* = 0.89) for O_3_, RR 1.24 (95% CI 0.90–1.71; *p* = 0.19) for NO_2_, and RR 1.18 (95% CI 0.66–2.13; *p* = 0.58) for PM_10_, respectively.

Tahara et al. (55) (352 participants) also investigated the link between air pollution and the risk of acute exacerbations of idiopathic interstitial pneumonias (AE-IIPs). Their study revealed positive associations between the average monthly exposure to NO, NO_2_, NOX, and PM_2.5_, and AE-IIPs. Specifically, for every 10 ppb increase, the aOR was 1.50 (95% CI 1.19–1.88; *p* = 0.001) for NO, 1.99 (95% CI 1.22–3.27; *p* = 0.006) for NO_2_, and 1.29 (95% CI 1.08–1.53; *p* = 0.004) for NOX. Additionally, for every 10 ug/m^3^ increase, the aOR was 2.88 (95% CI 1.69–4.91; *p* ≤ 0.001) for PM_2.5_. However, there was no significant association found between the average monthly levels of SO_2_, O_3_, CO, PM_10_, and AE-IIPs.

#### 3.4.4 Air pollution and lung function (baseline and decline in IPF, decline in fibrotic sarcoidosis patients)

Five studies (46, 48, 49, 58, 66) involving a total of 5,907 participants explored the relationship between air pollution and the decline in lung function among individuals with IPF or other fibrotic ILD. The pollutants investigated included PM_2.5_, PM_10_, O_3_, and NO_2_. Four studies found no significant association between PM_2.5_ levels and decline in FVC (46, 48, 49, 66). The sole study indicating an association was conducted by Goobie et al. (58) in patients with fILD. They observed that a 1 μg/m^3^ increase in the 5-year pre-censoring PM_2.5_ exposure, as assessed through adjusted meta-analysis across three cohorts, was linked to an additional 0.15% decrease in FVC percentage estimated per year (HR0.15, 95% CI -0.42 to 0.12, *p* = 0.29). Investigation of the constituents of PM_2.5_ revealed that higher exposures to sulfate, nitrate, and ammonium were correlated with a more accelerated annual decrease in the FVC percent predicted (SO_4_^2^: β −2.53, 95% CI -4.45 to −0.62, *p* = 0.01); (NO_3_-: β-1.72, 95% CI -2.86 to −0.58, *p* = 0.003); (NH_4_^+^: β-5.93, 95% CI -10.18to −1.69, *p* = 0.006).

The same study (58) also identified associations between PM_2.5_ and its constituents, particularly SO_4_^2^ and NH_4_^+^, and diffusing capacity of the lungs for carbon monoxide (DLco) decline. The adjusted meta-analysis showed non-significant association between total PM _2.5_ (HR: −0.05; 95% CI −0.31 to 0.21; *p* = 0.70), but significant association with its constituents SO_4_^2^ (β: −2.12; 95% CI −3.93 to −0.30; *p* = 0.02), and NH_4_^+^ (β: −4.66; 95% CI −8.77 to −0.54; *p* = 0.03). In the study by Zheng et al. (66), they also found a positive link between increased PM_2.5_ levels and DLco decline in IPF patients. Specifically, for each interquartile range (IQR) increase of 2.2 μg/m^3^ in PM_2.5_, there was an additional 0.9% predicted/year (95% CI −1.6 to −0.3) accelerated decline in DLco.

For PM_10_, Johannson et al. (46) did not observe any association between higher cumulative mean exposures or maximum exposures to air pollution and a more rapid decline in FVC or FEV1 over a duration of up to 40 weeks. In contrast to this, Winterbottom et al. (48) demonstrated that for every 5 μg/m^3^ increase in PM_10_ concentration, there was an additional decline of 46 mL per year in FVC (95% CI, 12–81 mL/y; *p* = 0.008). Neither NO_2_ (46, 66) nor O_3_ (46, 49), were found to be associated with a more rapid decline in lung function.

Among the five studies, two studies (6,708 participants) examined the association between air pollutants and baseline lung function (46, 58). Both studies identified a correlation between elevated air pollution levels and lower baseline lung functions. Johannson et al. (46) observed that increased mean levels of NO_2_(*p* = 0.03), PM_2.5_(*p* = 0.02), and PM_10_(*p* = 0.003) over the study period were significantly linked to reduced FVC % predicted. Goobie et al. (46) found that an increase in PM_2.5_ and its constituent mixture by one quantile was associated with decreased baseline FVC, specifically, higher MC is associated with a significant 3.38% decrease in the estimated baseline percentage of FVC (β-3.38; 95% CI −4.88 −1.87; *p* < 0.001) and a 3.64% decrease in the estimated baseline percentage of DLCO (β −3.64; 95% CI −4.61 to −2.66; *p* < 0.001).

#### 3.4.5 Air pollution and incidence of ILD

Three studies, which collectively involved nearly 10 million inhabitants from Italy (45), 379 IPF participants from Spain (54), and 433,738 participants from a biobank in the UK (61), investigated the connection between air pollution exposure and the incidence of IPF. They showed that NO_2_, PM_2.5_, and NOx were linked to an increased incidence of IPF. In the Italian study (45), the authors demonstrated that an increase of 10 μg/m^3^ NO_2_, was associated with an increase in the IPF incidence rate from 6.39% (95% CI −3.62–17.45) to 7.55% (95% CI −0.76–16.56%), depending on the season. In the UK study (61), the adjusted hazard ratios for IPF were aHR 1.11 (95% CI 1.03–1.19; *p* = 0.006) for NO_2_, aHR 1.07 (95% CI 1.01–1.13; *p* = 0.02) for NOx, and aHR 1.09 (95% CI 1.02–1.17, *p* = 0.01) for PM_2.5_, for each interquartile rise in these pollutants. Shull et al. (54) underscored the positive link between increased incidence of ILD and PM_2.5_, revealing that the highest PM_2.5_ concentrations, ranging from 20 to 24.6 μg/m^3^, were in areas of traffic congestion, industrial zones, the airport, and the ports of Barcelona. These areas, rather than the most densely populated ones, exhibited a higher aggregation of IPF cases. No significant association was discovered between the incidence of IPF and the concentrations of PM_10_ (45, 61) or O_3_ (45).

Two studies (4,883 participants) investigated the association between ambient air pollution and the likelihood of subclinical ILD or ILA (44, 50). Both studies observed a positive connection between NOx and elemental carbon (EC) exposure and the occurrence of ILD or ILA. Sack et al. (44) showed that for each 40-ppb increase in NOx concentration, the OR for ILD was 1.77 (95% CI 1.06–2.95, *p* = 0.03). In a separate study (50), an IQR difference in 5-year EC exposure of 0.14 μg/m^3^ was associated with an OR of 1.27 (95% CI 1.04–1.55, *p* = 0.02) for ILA. The same study also noted an OR of 1.33 (CI 1.00 to 1.76, *p* = 0.05) for the progression of ILA. Neither study showed an association between exposure to PM_2.5_ and ozone (O_3_) and the presence of ILA and Sack et al. study (44) showed no association between ILA incidence and NO_2_ exposure.

One case–control study (52) involving 2,525 participants examined the link between air pollution exposure and the development of ILD in patients with a variety of CTD diagnoses. The study revealed that individuals with CTD-ILD had somewhat lower average exposure to PM_2.5_, PM_10_, and SO_2_ when compared to patients with non-ILD CTD. However, these differences were not statistically significant. For every 10-ppb increase in O_3_ exposure, there was a decrease in the risk of developing ILD (aOR 0.51; 95% CI 0.33–0.79; *p* = 0.002).

#### 3.4.6 Air pollution and hospitalisation (IPF and RA-ILD)

Three studies (51, 59, 63) involving a total of 16,032 participants explored the impact of various air pollutants, namely NO_2_, O_3_, PM_2.5_, CO, SO_2_, NO_X_, on hospitalisation incidence in patients with IPF. In all three studies, the investigation focused on the exposure to NO_2_ and O_3_, and consistently revealed a positive correlation between the mean concentration of NO_2_, whether it was from short-term or long-term exposure, and the incidence of hospitalization. Nevertheless, the findings regarding O_3_ were inconclusive. Mariscal-Aguilar et al. (63) found an association between elevated mean O_3_ levels during the month preceding admission and a heightened rate of hospital admissions. In contrast, Dale et al. (51) did not observe a statistically significant association in their two-pollutant model. Liang et al. (59) showed a positive association with short-term increased level of O_3_ exposure in men and hospital admissions (RR: 1.045; 95% CI 1.000–1.092; *p* = 0.05) per IQR increase of 85 μg/m^3^, but not with average O_3_ exposure over the 30 days period before admission.

Two of these studies also investigated PM_2.5_, Dale et al. (51) found a positive association (RR 1.29, 95% CI 1.09–1.54, *p* = 0.003) between short-term elevated average level of PM_2.5_ (for an IQR Increase) and an increased incidence of hospitalisation. Liang et al. (59) also observed a positive association with short-term PM_2.5_ exposure (RR: 1.049, 95% CI 1.024–1.074, *p* = 0.0001) for lag0 and RR 1.031 (95% CI 1.007–1.056; *p* = 0.01) for a moving average 0-1 days, both per IQR of 72 μg/m^3^. However, this association was not evident for average PM_2.5_ exposure over 0–30 days. PM_10_ level was found to be one of the strongest independent pollutants associated with increased hospitalisation (51) (RR 1.31; 95% CI 1.12–1.53; *p* < 0.001), but less statistically significant in Liang et al.’s study (per 86 μg/m^3^: RR 1.021, 95% CI 0.994–1.049, *p* = 0.13) (59).

CO exposure was initially found to have a positive association with hospitalisation (51), but this association was lost when using two-pollutant models. In contrast, Mariscal-Aguilar et al. (63) did not find any association between incidence of hospitalisation and CO levels. Regarding SO_2_ exposure, Dale et al. (51) initially demonstrated a positive correlation between elevated SO_2_ concentration and hospitalization during univariable analysis, with a relative risk (RR) of 1.20 (95% CI 1.07–1.34, *p* < 0.05), but this relationship lost significance when two-pollutant models were applied. Liang et al. (59) identified an association between short-term SO_2_ exposure and hospitalization in men. Elevated exposure to NO_X_ concentrations, as examined by Mariscal-Aguilar et al. (63), was significantly linked to the incidence of hospitalization in IPF patients for respiratory causes, particularly with exposures spanning 6, 12, and 36 months.

A single study focusing on RA-ILD patients (221 participants) (60), found that per 10 μg/m^3^ increase in PM_2.5_, PM_10_, SO_2_, and NO_2_ were each significantly associated with increased monthly hospitalisation admissions. The study showed both higher mean and cumulative concentrations of O_3_, for every 10 mg/m^3^ increase in the 0–2-month lag, displayed an inverse relationship (Excess risk −0.304 to −0.393; and − 3.383 to −0.406) implying that higher levels of O_3_ were linked to lower rates of hospitalisations for RA-ILD.

#### 3.4.7 Air pollution and supplemental oxygen requirement

One study (135 patients with IPF) (48) examined the link between air pollution and supplemental oxygen requirement during the 6-min walk test (6MWT) in IPF patients. The findings revealed an association between higher mean level of PM_2.5_ and an increase in oxygen requirement to maintain saturation > =88% during the 6MWT, with each 5 μg/m^3^ increase in PM_2.5_ exposure corresponding to a 1.15 (95% CI, 0.03–2.26; *p* = 0.044) increase in O_2_ requirement.

#### 3.4.8 Air pollution and development of chronic respiratory failure in IPF

In a study involving 69 individuals with IPF (63), researchers examined the relationship between air pollution and the occurrence of chronic respiratory failure, as defined by baseline arterial blood gases (ABG), measured while they were breathing room air, with a partial pressure of oxygen (PO_2_) below 60 mmHg (63). Elevated exposure to CO, NO_2_, and NOx were found to be significantly associated with a higher risk of developing chronic respiratory failure. Specifically, for every 0.1 mg/m^3^ increase in CO, the OR was 1.62 (1.11–2.36) (*p* = 0.01) over a 3-month period and 1.84 (1.1–3.06) during the 6 months leading up to the event. For each 10 μg/m^3^ increase in NO_2_ and NO_x_: the OR for NO_2_ was 1.65 (1.01–2.66) (*p* = 0.04) at 6 month prior to the event. For NOx, the OR was 1.12 (1.01–1.23) (*p* = 0.03) at 3 months and 1.20 (1.03–1.38) (*p* = 0.01) at 6 months of exposure before the event.

#### 3.4.9 Air pollution and questionnaire outcomes in fibrotic sarcoidosis patients

In a study involving 16 participants with fibrotic sarcoidosis (49), correlations between short-term air pollution exposure and health outcomes were evaluated through validated health related QoL questionnaires. With each IQR increase in the 14-day average of PM_2.5_ levels, a decrease in general and lung-specific health status was observed as measured by the King’s Sarcoidosis Questionnaire (KSQ) (General: −6.60, 95% CI −12.51 to −0.68,; Lung-specific: −6.91, 95% CI −12.73 to −1.09) The study did not find any association between short-term O_3_ exposure and respiratory symptoms or QoL metrics, using the Saint George Respiratory Questionnaire (SGRQ), Leicester cough questionnaire (LCQ), and KSQ.

#### 3.4.10 Air pollution and surrogate markers

DNA methylation (DNAm) can be inherited and influenced by air pollution in adults (67, 68). Aberrant DNAm patterns lead to reduced DNA stability and changes in gene expression and mutations, which can contribute to health problems (67, 68). Goobie et al. examined 1,059 participants from two groups of patients with IPF: 313 from the Simmons cohort and 746 from the PFF cohort (62). They investigated the association between PM_2.5_ (and its constituent components) with global DNAm (%5 mC) in patients with IPF. This study found an association between monthly mean levels of PM_2.5_ and its constituents’ exposure and increased level in %5 mC (β = 0.02, 95%CI 0.0003–0.05, *p* = 0.047) over the 3-month period before sampling. The results in the 1-year period before sampling were consistent but did not reach statistical significance in both cohorts (Simmons cohort: β = 0.03, 95%CI −0.004 to 0.06, *p* = 0.09; PFF cohort: *β* = 0.03, 95%CI −0.007 to 0.06, *p* = 0.12). Increased levels of sulfate, nitrate, ammonium, and black carbon components were also linked to elevated %5 mC across multiple models.

One study of 118 participants with IPF (56) examined the relationship between prolonged exposure to air pollution and the presence of serum inflammatory markers. The study found that an increase of 10 μg/m^3^ in the mean O_3_ level from the previous year was positively correlated with the percentage change in IL-4 (an inflammatory mediator) among AE-IPF patients (*p* = 0.014). Conversely, PM_2.5_, PM_10_, and NO_2_ levels showed an inverse association with the percentage changes in IL-4 (*p* = 0.003, *p* = 0.003, *p* = 0.032) and osteopontin (*p* = 0.013, *p* = 0.013, *p* = 0.085) among the same group of patients.

## 4 Discussion

In this comprehensive systematic review, of the link between air pollution and ILD, including IPF we evaluated 24 studies which met the inclusion criteria. The pollutants under investigation include PM_2.5_, PM_10_, O_3_, NO_2_, CO, SO_2_, NOx, and NO. All of these pollutants were considered in the assessment of AE-IPF risk. The meta-analyses performed showing a significant association between AE-IPF and PM_2.5_, while the association between O_3_, NO_2_, PM_10_, and AE-IPF risk remained uncertain.

### 4.1 Particulate matter (PM_2.5_ and PM_10_)

Particulate matter, a blend of solid particles and liquid droplets present in the atmosphere, includes PM_2.5_ and PM_10_ (69). PM_2.5_ is characterized as fine inhalable particles, typically measuring 2.5 micrometres or less in diameter, while PM_10_ is described as inhalable particles, generally having diameters of 10 micrometres or less (69). PM_2.5_ arises as a result of sophisticated atmospheric interactions involving combustion and the conversion of gases into particles (70, 71). It originates predominantly from a range of sources, encompassing the combustion of coal, oil, gasoline, diesel, and wood, as well as high-temperature industrial operations including those conducted in smelters and steel mills (71). Additionally, vehicle exhaust emissions and the burning of biomass contribute significantly to the production of PM_2.5_ (71, 72). PM_10_ is produced through the fragmentation of larger solid particles, the presence of biological materials like pollen grains, and the dispersion of wind-blown dust from agricultural areas, soil surfaces, unpaved roads, and dust generated by traffic (70). Additionally, it can originate from non-combustible substances such as fly ash (70).

Particulate matter has the potential to lead to severe health issues. PM_2.5_ has the tendency to settle deep within the respiratory airways, potentially penetrating into the alveoli, in some cases, being absorbed into the bloodstream (69, 70). This makes it particularly challenging to eliminate from the body. PM_10_ comprises particles that are larger in size, yet they are still small enough to penetrate into the lungs (70). Elevated levels of both PM_2.5_ and PM_10_ have been associated with higher rates of all-cause mortality, cardiovascular mortality, respiratory mortality, and mortality due to lung cancer (73).

Most of the included studies in this analysis evaluated PM_2.5_and PM_10._ It is interesting that both PM_2.5_ and PM_10_ were associated with increased hospital admissions without apparent influence on the progression of IPF. One possible explanation for this finding lies in the temporal dynamics of PM_2.5_ and PM_10_ exposure. PM_2.5_ infiltrates alveoli (69, 70), while PM_10_ deposits in the upper respiratory tract (25), both capable of precipitating acute respiratory exacerbations necessitating hospitalization. However, it appears that these exposures may not have a lasting impact on the lung function decline (FVC%), which serves as an indicator of disease progression in the studies under consideration (47, 64, 66). This is consistent with the findings that there was no significant association between PM_2.5_ (46, 48, 49) or PM_10_ (46, 48, 66) with lung function decline.

### 4.2 Ozone (O_3_)

O_3_ can be categorized into two types: beneficial stratospheric ozone and detrimental ground-level O_3_ (74). Stratospheric ozone serves as a shield against the harmful ultraviolet radiation from the sun, protecting living organisms (74). Conversely, ground-level O_3_ has been linked to elevated all-cause mortality rates, respiratory mortality, hospital admissions for asthma-related issues, and visits to emergency rooms (73).

Ground-level O_3_ is not directly released into the atmosphere; rather, it forms through a complex sequence of reactions involving oxides of nitrogen (NOx) and volatile organic compounds (VOC) when exposed to heat and sunlight (70, 74). VOCs are compounds that characterized by their high vapor pressure, and their primary sources predominantly include emissions from industrial and agricultural activities (25). Additionally, VOCs can stem from sources such as wildfires, vegetation, animals, and vehicles (25). Due to the reaction mechanism with heat and sunlight, O_3_ tends to attain elevated, unhealthy levels primarily during hot, sunny days in urban settings (25, 74).

Our findings regarding O_3_ are varied. Interestingly, an inverse correlation was found between O_3_ concentration and hospitalization incidence in RA-ILD and the development of ILD in patients with CTDs (52). This interesting outcome warrants careful interpretation because the protective influence of O_3_ against ILD risk appears to be consistently observed solely in patients with systemic lupus erythematosus (SLE), rather than in those with other CTDs (52). Increased O_3_ levels necessitate a reaction mechanism involving heat and sunlight (25, 74). Given that photosensitivity is a well-established exacerbating factor for SLE (75), it implies that individuals with SLE may opt to stay indoors to avoid direct sunlight. This behaviour could potentially explain why heightened levels of O_3_ appear to correlate with a reduced risk of ILD among SLE patients, as they may, in fact, experience lower O_3_ exposure.

### 4.3 Nitrogen oxides (NOx), nitric oxide (No) and nitrogen dioxide (NO_2_)

NOx encompasses a collection of exceedingly reactive gases, consisting of varying proportions of nitrogen and oxygen, with the most common components being NO_2_, NO, and nitrous oxide (N_2_O) (76). The primary sources of NOx emissions encompass motor vehicles, electric utilities, and a diverse array of industrial, commercial, and residential activities involving fuel combustion (76).

This review encompassed an exploration of NOx in four distinct studies. Positive correlations were identified for all health outcomes under investigation. Remarkably, the findings indicated significant links between NOx and the occurrence of subclinical ILD (44), heightened mortality rates in individuals with IPF (63), an increased risk of experiencing AE-IPF and AE-IIPs (55), elevated occurrences of IPF (61), higher hospitalization rates among those with IPF (63), and a hastened progression towards chronic respiratory failure in this population (63). Although each health outcome was examined in separate studies, it’s noteworthy that NOx consistently showed a positive association with these risks. This recurring pattern strongly suggests that NOx may play a significant role in exacerbating these health issues in ILDs. This finding underscores the importance of further research and attention to the potential health impacts of NOx exposure and calls for comprehensive efforts to mitigate NOx emissions and protect public health.

Within industrial combustion processes, NO predominates, often surpassing NO_2_ in both quantity and concentration (76, 77). However, the majority of NO molecules eventually undergo oxidation, transforming into the more toxic NO_2_ gas (77). NO is harmful to human health and can result in eye and throat irritation, chest tightness, nausea, headaches, and a gradual decline in physical strength (76). NO was assessed in a single study, which revealed that an increased concentration of NO was significantly linked to an elevated risk of AE-IPF and AE-IIPs (55). Elevated levels of nitric oxide are recognized for their inflammatory characteristics (78), potentially fostering an inflammatory setting that could trigger exacerbation events in interstitial lung diseases. However, it is crucial to note that this finding originates from a solitary study, emphasizing the necessity for further investigations to validate and substantiate these results.

NO_2_ is a gas and strong oxidant (70). NO_2_, in conjunction with other NOx components, engages in chemical reactions with the previously mentioned VOCs in the air, leading to the formation of O_3_ (70, 79). Additionally, NO_2_ serves as a crucial precursor to other secondary pollutants, including organic, nitrate, and sulfate particles, which are measured as part of PM_10_ or PM_2.5_ particulate matter (70). NO_2_ predominantly enters the atmosphere through the combustion of fuel (79). It arises from the emissions produced by vehicles such as cars, trucks, and buses, as well as from power plants and off-road machinery (79). Elevated levels of NO_2_ are associated with increased rates of all-cause mortality, respiratory mortality, hospital admissions related to asthma, and visits to the emergency room (73). It’s interesting to note that NO_2_, which is a component of NOx, appears to have distinct effects on various health outcomes. Our findings found that NO_2_ exhibited a significant positive correlation with an increased incidence of hospitalization in IPF (51, 59, 63) and RA-ILD (60), a heightened risk of AE-IIPs (55), lower lung function (46), and the development of chronic respiratory failure (63). However, no associations were detected between NO_2_ and the severity at diagnosis and progression at 24 months of SSc-ILD (65), lung function decline in IPF (46), or the incidence of subclinical ILD/ILAs (44).

### 4.4 Carbon monoxide (CO)

CO is a toxic gas that is colourless, has no odour or taste (26). It is generated when carbonaceous fuels such as wood, gasoline, coal, natural gas, and kerosene undergo incomplete combustion (26). CO exhibits a greater affinity for binding with haemoglobin, potentially resulting in diminished oxygen transport to vital organs (70). Elevated levels of CO are linked to a rise in hospital admissions and visits to the emergency room attributable to ischemic heart disease (73).

Our review found that CO was positively associated with the development of chronic respiratory failure (63) and had mixed results in terms of its relationship with mortality in IPF (53, 63). The presence of a positive association with the onset of chronic respiratory failure suggests a potential mechanism wherein increased levels of CO in the ambient air may bind to haemoglobin and lead to a decrease in the PO_2_ (63).

### 4.5 Sulfur dioxide (SO_2_)

SO_2_ is an invisible gas that readily dissolves in water (70). Its primary source arises from the combustion of fossil fuels conducted by power plants and various industrial activities (70, 80). Smaller contributions to SO_2_ emissions come from natural occurrences such as volcanic activity, as well as vehicles, locomotives, ships, and heavy machinery utilizing high-sulfur content fuels (70, 80). Increased SO_2_ concentrations have been reported as contributing to heightened rates of all-cause mortality, hospital admissions for asthma-related issues, visits to emergency rooms, and respiratory mortality (73).

This review found a positive link between SO2 and hospitalization in RA-ILD (60). However, for hospitalization incidence in IPF patients, the findings were mixed (51, 59). No significant associations were identified between SO_2_ and mortality in IPF (53), the risk of AE-IPF (43, 55), or the risk of AE-IIPs (55). The diversity in these findings may stem from varying geographical locations and levels of exposure. Two studies showing a positive association both reported a higher mean SO_2_ concentration than the other four studies, with a mean SO_2_ concentration of 28.15 ± 19.08 μg/m3 (60) and 23.6 μg/m3 (51).

### 4.6 Limitations

In this review we included a substantial number of participants. Nevertheless, it’s imperative to acknowledge certain limitations. Firstly, the studies encompassed in this review only spanned 13 countries or regions, which represents a relatively small portion of the global population. Secondly, a limited number of studies examined each specific health outcome, potentially not providing a comprehensive reflection of the true outcomes. It’s crucial to be caution when interpreting the meta-analysis results concerning the association between air pollutants and the risk of AE-IPF, primarily due to the significant heterogeneity observed, ranging from 67 to 88% across the studies, except for PM_2.5_(6%). Additionally, we acknowledge that potential confounding factors such as temperature, humidity, seasons, and occupational environments can impact the results. Socioeconomic status can also play a significant role in ILD incidence and outcomes. IPF patients with lower economic status experienced poorer survival outcomes, despite adjusting for occupational exposure and geographical origin (81).

Another significant constraint is that the various studies opted for different outcome measures, making it impossible to conduct a meta-analysis encompassing all the desired outcomes. Moreover, a notable limitation stems from the substantial variation in population sizes observed across studies, spanning from 16 participants to 10 million. Lastly, our study was not pre-registered with PROSPERO.

## 5 Conclusion

This comprehensive review highlights the need for further research efforts to gain a deeper understanding of the complex relationship between air pollution and ILDs. Future investigations should endeavour to incorporate real-time data collection methodologies, encompassing factors including the duration spent indoors and in traffic, which could provide valuable insights into individual exposure patterns. Additionally, there is a need for the standardization of the parameters being measured across various studies, ensuring consistency and comparability of findings. Moreover, employing multi-pollutant models in future research would be beneficial in resolving the complex interaction of various pollutants in affecting ILD outcomes.

The available data hints at the likely benefit for public health intervention. Specifically, there is compelling evidence to suggest that reducing levels of PM_2.5_ in the atmosphere could potentially lead to a reduction in the frequency and severity of acute exacerbations (AE) in individuals with ILDs. This finding bares significance, as AE in ILD patients has been consistently linked to high mortality rates, frequent hospitalizations, and elevated healthcare costs (82). The potential benefits from the interventions could not only improve individual health outcomes but also alleviate the burden on healthcare systems and resources.

## Data availability statement

The original contributions presented in the study are included in the article/Supplementary material, further inquiries can be directed to the corresponding author.

## Author contributions

DL: Conceptualization, Data curation, Formal analysis, Investigation, Methodology, Project administration, Writing – original draft. CF: Supervision, Validation, Writing – review & editing. LT: Supervision, Writing – review & editing. LK: Conceptualization, Supervision, Writing – review & editing. TC: Conceptualization, Supervision, Writing – review & editing.
