# Supplementary material for: The impact of air pollution on interstitial lung disease: a systematic review and meta-analysis
Source: Front Med (Lausanne). 2024 Jan 17;10:1321038. doi: 10.3389/fmed.2023.1321038 (PMC10827982; doi:10.3389/fmed.2023.1321038)
Supplement: Supplementary file 1 [file Table_1.docx]

**Supplementary Table S1: Impact of air pollutants on ILDs**

| **Themes** | **Studies** | **Air Pollution Metrics** | **Outcome findings (risk/incidence/occurrence/ odds)** | | | **Comments** |
| --- | --- | --- | --- | --- | --- | --- |
|  |  |  | Positive | No Impact | Inverse |  |
| Mortality in IPF or fILD | Johannson, 2014 (43) | O_3_ and NO_2_ |  | 🗸 |  | Cumulative mean O_3_ HR 1.03(95% CI 0.90-1.17); NO_2_ HR 0.97 (95% CI 0.86–1.10). Maximum O_3_ HR 1.02 (95% CI 0.90–1.16); NO_2_ HR 1.06 (95% CI 0.93–1.20) |
|  | Sesé, 2018 (47) | PM_10_ and PM_2.5_ | 🗸 |  |  | Per 10 µg/m³ increase:PM_10_ (HR=2.01, 95% CI 1.07 to 3.77) (p=0.03); PM_2.5_ (HR=7.93, 95% CI 2.93 to 21.33) (p<0.001). |
|  |  | NO_2_ and O_3_ |  | 🗸 |  | Per 10 µg/m³ increase: NO_2_ HR1.01 (95% CI 0.79 to 1.29) (p=0.90); O_3_ HR0.89 (95% CI 0.66 to 1.18) (p=0.43) |
|  | Mariscal-Aguilar, 2021 (53) | CO | 🗸 |  |  | For each increase of 0.1 mg/m^2^: OR 2.45, 95% CI 1.39–4.56 (p= 0.005) |
|  |  | PM_2.5_, PM_10_, NO_2_, SO_2_ and O_3_ |  | 🗸 |  | Per each 5ug/m^2^ increase:NO_2_: OR 1.13, 95% CI, 0.84–1.50; PM_2.5_: OR 1.12, 95% CI, 0.30–4.23; PM_10_: OR 0.78, 95% CI, 0.37–1.66; O_3_: OR 0.90, 95% CI, 0.75– 1.09]; SO_2_: OR 1.19, 95% CI, 0.37–3.82 |
|  | Yoon, 2021 (57) | NO_2_ | 🗸 |  |  | Per 10ppb increase in NO_2_: HR 1.172, 95% CI 1.030–1.344; p=0.016 |
|  |  | PM_10_ |  | 🗸 |  | Per 10 µg/m³ increase: Figure Forest plot available, detailed statistic data not available. Described as "PM_10_ was not associated with IPF mortality in all patients and in subgroups stratified by age or sex." |
|  | Goobie, 2022 (58) | PM_2.5_ and its constituents: higher SO_4_^2−^, NO_3_ ^−^, and NH_4_^+^ levels | 🗸 |  |  | An exposure of 8 μg/m³ or higher in the 5-year precensoring, meta-analysis: PM_2.5_(HR, 1.18 [95% CI, 1.02-1.37]; p =0.03; SO42- β 8.02 (95% CI, 0.52 to 122.63) p= 0.13; NO_3_ β-3.78 (95% CI,2.30 to 6.20) p<0.001; NH_4_+ β 50.99 (95% CI 2.46 to 1056.64) p= 0.01 |
|  | Mariscal-Aguilar, 2023 (63) | NO_2_, O_3_, and NOx | 🗸 |  |  | Increases in the averages of NO_2_, O_3_, and NOx were significantly associated with an increase in the probability of death. in patients with an exposure to these pollutants of 1 and 3 months in the case of O_3_ and of 12 and 36 months in the case of NO_2_ and NOx. |
|  |  | CO |  | 🗸 |  | No specific data |
| Disease Progression of IPF | Sesé, 2018 (47) | NO_2_, O_3_, PM_10_ and PM_2.5_ |  | 🗸 |  | Per 10 µg/m³ increase: NO_2_ HR1.09 (95% CI 0.85 to 1.40) (p=0.52); O_3_ HR 1.06 (95% CI 0.74 to 1.54) ( p= 0.72);PM_10_ HR 1.03 (95% CI 0.51 to 2.08) (p=0.92);PM_2.5_ HR1.89 (95% CI 0.68 to 5.23)(Pp=0.22) |
|  | Yoon, 2023 (64) | NO_2_ | 🗸 |  |  | A 10-ppb increase in NO_2_ concentration a 10.5% increase in the risk of progression (HR 1.105; 95% CI 1.000–1.219) (p = 0.048) |
|  |  | PM_10_ |  | 🗸 |  | PM_10_ was not associated with disease progression in both unadjusted and adjusted models |
|  | Zheng , 2023 (66) | PM_2.5_ and NO_2_ |  | 🗸 |  | PM_2.5_ (HR 1.14, 95% CI 0.84 to 1.53) (p=0.396); NO_2_ (HR 1.05, 95% CI 0.82 to 1.34)(p=0.725) |
| The risk of AE-IPF | Johannson, 2014 (43) | O_3_ and NO_2_ | 🗸 |  |  | O_3_(mean)HR 1.57,95% CI 1.09–2.24, p=0.01; (Maximum)HR 1.42, 95% CI 1.11–1.82, p=0.01; and(exceedances) HR 1.51, 95% CI 1.17–1.94,p=0.002; NO_2_ (mean)HR 1.41, 95% CI 1.04–1.91,p=0.03;(Maximum) HR 1.27, 95% CI 1.01–1.59,p=0.04; and (exceedances) HR 1.20, 95% CI 1.10–1.31,p<0.001 |
|  |  | PM_10_, SO_2_ and CO |  | 🗸 |  | CO (mean) HR1.22 (95% CI 0.97–1.55) p= 0.09;(Maximum)HR1.07 (95% CI 0.77–1.47) p=0.7;PM 10 (Mean)HR1.08 (95% CI 0.77–1.51) p=0.66; (Maximum)HR1.08 (95% CI 0.76–1.52) p=0.68; (exceedances) HR1.06 (95% CI 0.83–1.36) p=0.62; SO_2_ mean HR 1.04 (95% CI 0.73–1.47) p=0.84; Maximum HR 1.03 (95% CI 0.81–1.32) p=0.79 |
|  | Sesé, 2018 (47) | O_3_ | 🗸 |  |  | Per 10 µg/m³ increase: O_3_ HR 1.47 (95% CI 1.13 to 1.92) (p=0.005) |
|  |  | NO_2_, PM_2.5_, and PM_10_ |  | 🗸 |  | Per 10 µg/m³ increase: NO_2_ HR 0.92 (95% CI 0.68 to 1.24) (p=0.584);PM_10_ HR 0.80 (95% CI 0.52 to 1.27)(p=0.347);PM_2.5_ HR1.29 (95% CI 0.65 to 2.57)(p=0.347) |
|  | Tahara, 2021 (55) | NO, NO_2_, NOx, PM_2.5_ | 🗸 |  |  | Per 10ppb increase: NO: OR 1.46 (95% CI 1.11–1.93) p=0.008; NO_2_ OR1.71 (95% CI 0.89–3.25) p= 0.105; NO_X_ OR 1.24 (95% CI 0.99–1.53) p=0.052; per 10 ug/m³ increase: PM_2.5_ was 2.56 (95% CI 1.27–5.15)p=0.009; |
|  |  | SO_2_, O_3_, CO, PM_10_ |  | 🗸 |  | Per 10ppb increase: SO_2_ OR 0.35 (95% CI 0.03–3.88) p=0.39; O_3_ OR 0.99 (95% CI 0.60–1.64) p=0.983; CO OR 1.01 (95% CI 0.99–1.02 p=0.52; per 10 ug/m³ increase: PM_10_ OR 1.04 (95% CI 0.55–1.99) p= 0.90 |
|  | Tomos, 2021 (56) | NO_2_, PM_2.5_, and PM_10_ | 🗸 |  |  | Per 10 μg/m³ increase in previous year mean level.NO_2_ (OR=1.52, 95%CI:1.15–2.0, p=0.003), PM_2.5_ (OR=2.21, 95%CI:1.16–4.20, p=0.016) and PM_10_ (OR=2.18, 95%CI:1.15–4.15, p=0.017) |
|  |  | O_3_ |  | 🗸 |  | Per 10 μg/m³ increase in previous year mean level: O_3_ OR 0.60 (95%CI:0.43 to 0.86) p=0.005 |
| The risk of AE-IIPs | Tahara, 2021 (55) | NO, NO_2_, NOx, PM_2.5_ | 🗸 |  |  | Per 10ppb increase: NO aOR1.50 (95% CI 1.19–1.88; p=0.001), NO_2_ aOR 1.99 (95% CI 1.22–3.27; p=0.006), NOX aOR 1.29 (95% CI 1.08–1.53; p=0.004), and per 10 ug/m³ increase: PM_2.5_ aOR 2.88 (95% CI 1.69–4.91; p≤0.001). |
|  |  | SO_2_, O_3_, CO, PM_10_ |  | 🗸 |  | Per 10ppb increase:SO_2_ aOR 1.30 (95% CI0.19–8.69; p=0.79), O_3_ aOR 0.90 (95% CI 0.64–1.27; p=0.55), CO aOR 1.01 (95% CI0.99–1.03; p=0.16), per 10 ug/m³ increase: PM_10_ aOR 1.11 (95% CI 0.70–1.78; p=0.65) |
| Lung Function decline in IPF or fibrotic interstitial lung disease (fILD) | Johannson, 2018 (46) | O_3_, NO_2_, PM_2.5_, and PM_10_ |  | FVC |  | Neither higher cumulative mean exposures nor maximal exposures to air pollution were associated with more rapid decline in FVC or FEV1(for up to 40 weeks) |
|  | Winterbottom, 2018 (48) | PM_10_ | FVC |  |  | Significant association: each 5μg/m³ increase in PM_10_ corresponding with an additional 46 cc/y decline in FVC (95% CI, 12-81 cc/y)(P = .008) |
|  |  | PM_2.5_ |  | FVC |  | β-coefficient 34(95% CI, –60 to 127) p= .48 |
|  | Goobie, 2022 (58) | PM_2.5_ and its constituents: SO_4_^2−^, NO_3_ ^−^, and NH_4_^+^ | FVC |  |  | An increase of 1 μg/m³ in the 5-year precensoring, Adjusted meta-analysis: PM 2.5 HR-0.15 (95% CI -0.42 to 0.12) (p=0.29);SO42- β -2.53 (95% CI -4.45 to -0.62)(p= 0.01); NO_3_- β-1.72(95% CI -2.86 to -0.58) (p= 0.003) ; NH4+ β-5.93(95% CI -10.18to -1.69)(p= 0.006 ) |
|  |  | PM_2.5_ and its constituents: SO_4_^2−^, and NH_4_^+^ | DLco |  |  | An increase of 1 μg/m³ in the 5-year precensoring, Adjusted meta-analysis: PM 2.5 HR-0.05 (95% CI -0.31 to 0.21) (p=0.70); SO42- β-2.12 (95% CI-3.93 to -0.30) (p=0.02); NH_4_+β-4.66 (95% CI -8.77 to -0.54) (p= 0.03) |
|  | Zheng, 2023 (66) | Living near a major road and increased PM_2.5_ | DLco | FVC |  | Each IQR of 2.2 μg/m³ increase in PM_2.5_: FVC (-0.1% predicted/year, 95% CI -0.6 to 0.4); 0.9% predicted/year (95% CI -1.6 to -0.3) faster decline in DLco |
|  |  | NO_2_ |  | DLco & FVC |  | IQR of 3.3 ppb increase in NO_2_: FVC (-0.3% predicted/year, 95% CI -0.7 to 0.2); -0.2% predicted/year (95% CI -0.7 to 0.4) |
|  | Pirozzi, 2018 (49) | PM_2.5_ and O_3_ |  | 🗸 |  | 14-day average PM_2.5_, FVC %change<0.01 (95% CI −2.25 to 2.31); O_3_ FVC % change 0.80 per IQR increase, (95% CI −3.13 to 4.88) |
| IPF incidence/prevalence | Conti, 2018 (45) | NO_2_ | 🗸 |  |  | 10 μg/m³ increase in NO_2_ concentration, increase between 6.39% (95% CI −3.62–17.45) and 7.55% (95% CI −0.76–16.56%) in IPF incidence rate, depending on season. |
|  |  | PM_10_ and O_3_ |  | 🗸 |  | Mixed results in different models |
|  | Shull, 2021 (54) | PM_2.5_ | 🗸 |  |  | Higher PM 2.5 concentrations (annual mean>10μg/m³) correlated with higher aggregation of IPF cases. |
|  | Cui, 2023 (61) | NO_2_, NOx and PM_2.5_ | 🗸 |  |  | Per each IQR increase, NO_2_ HR1.11 (95% CI 1.03–1.19); NOX HR 1.07 (95% CI 1.01– 1.13); PM_2.5_ 1.09 (95% CI 1.02–1.17) |
|  |  | PM_10_ |  | 🗸 |  | Per each IQR increase, PM_10_ HR 1.00 (95% CI 0.96–1.06) |
| Lower Lung Function baseline | Johannson, 2018 (46) | NO_2_, PM_2.5_, and PM_10_ | FVC |  |  | Lower mean FVC % predicted was consistently associated with increased mean exposures to PM_10_ in the 2 to 5 weeks preceding clinical measurements (range, –0.46 to –0.39 [95% CI, –0.73 to –0.13]; P < .005); Lower mean FVC % predicted over the study period was inversely related to mean levels of NO_2_ (–0.45 [95% CI, –0.85 to –0.05]; P = .03), PM_2.5_(–0.45 [95% CI, –0.84 to –0.07]; P = .02), and PM_10_ (–0.57 [95% CI, –0.92 to –0.21];P = .003) |
|  | Goobie, 2022 (58) | PM_2.5_ and its constituent mixture | DLco & FVC |  |  | 5-year preenrollment Adjusted meta-analysis: a 1-quantile increase in the constituent mixture was associated with a 3.38% lower estimated percentage baseline FVC (95% CI, −4.88 to −1.87; p < .001); each 1-quantile increase in constituent mixture was associated with a 3.64% lower estimated baseline percentage DLCO (95% CI, −4.61 to −2.66; p< .001; I2 = 9%) |
| The incidence of Subclinical ILD/ILAs | Sack, 2017(44) | NOx | 🗸 |  |  | Per 40ppb increment in NOx, OR1.77 (95%CI 1.06 to  2.95, p-value 0.03) |
|  |  | PM_2.5_, NO_2_, or O_3_ |  | 🗸 |  | ILAs were not associated with ambient PM_2.5_, NO_2_, or O_3_ concentrations |
|  | Rice, 2019 (50) | Elemental carbon (EC) | 🗸 |  |  | IQR difference in 5-year EC exposure of 0.14 µg/m³ ILA OR 1.27 (95% CI 1.04 to 1.55), ILA progression OR 1.33 (95% CI 1.00 to 1.76) |
|  |  | PM_2.5_ and O_3_ |  | 🗸 |  | PM_2.5_ ILA OR1.02 (95% CI 0.85 to 1.23), ILA progression OR 1.14 (95% CI 0.87 to 1.50); O_3_ ILA OR 0.91 (95% CI 0.78 to 1.06), ILA progression 0.97 (95% CI 0.78 to 1.21). |
| Hospitalisation incidence with IPF | Dales, 2020 (51) | PM_2.5_, NO_2_ and PM_10_ | 🗸 |  |  | Per an IQR increase, PM_2.5_ RR1.29 (95% CI 1.09-1.54); NO_2_ RR1.44 (95% CI1.09-1.92); PM_10_ RR 1.31(95% CI 1.12-1.53) |
|  |  | CO, O_3_, and SO_2_ |  | 🗸 |  | CO, O_3_ and SO_2_ effect lost significance in Two-Pollutant Models |
|  | Liang, 2022 (59) | PM_2.5_ | At lag0 & moving averages 0–1 day | Average exposure over 0-30days |  | Per IQR (72 μg/m³) higher, PM_2.5_ RR 1.049 (95% CI 1.024–1.074) (lag 0) RR 1.031 (95% CI 1.007–1.056) (moving average 0-1days); moving average 0-30, null association with  PM_2.5_ (RR: 0.994, 95% CI 0.962–1.027) |
|  |  | O_3_, NO_2_, &SO_2_ | At lag0 | Average O_3_ exposure over 0-30days |  | For each IQR (30 μg/m³) higher of NO_2_ at lag0 (RR: 1.064, 95% CI 1.023–1.107) in men only; SO_2_ at lag0 (RR: 1.023 (95% CI 1.002–1.045) per IQR (15 μg/m³) higher; at lag0 for O_3_ (RR: 1.045, 95% CI 1.000–1.092 per IQR (85 μg/m³) higher; |
|  |  | PM_10_, NO_2_, and SO_2_ | Average exposure over 0-30days |  |  | PM_10_ (RR per 86 μg/m³: 1.021, 95% CI 0.994–1.049) NO_2_ (RR per 30 μg/m³: 1.029, 95% CI 0.999–1.060), and SO_2_ (RR per 15 μg/m³: 1.060 (95% CI 1.025–1.097) |
|  | Mariscal-Aguilar, 2023 (63) | NO_2_, O_3_, and NOx | 🗸 |  |  | The increase in the averages of NO_2_, O_3_, and NOx were significantly associated with an increase in the probability of hospital admission due to respiratory causes |
|  |  | CO |  | 🗸 |  | No specific data |
| Hospitalisation incidence with RA-ILD patients | Liu, 2022 (60) | PM_2.5_, PM_10_, SO_2_, and NO_2_ | 🗸 |  |  | Per 10µg/m³ increase, monthly admissions of RA-ILD PM_2.5_ ER 0.875(95% CI: 0.375–1.377%), PM_10_ ER 0.548 (95% CI: 0.148– 0.949%), SO_2_ ER 1.968 (95% CI: 0.869–3.080%), and NO_2_ ER 1.534 (95% CI: 0.305–2.778%) |
|  |  | O_3_ |  |  | 🗸 | Per 10µg/m³ increase, monthly admissions O_3_ ER -0.304 (95% CI: -0.019, 0.589) |
| The severity at diagnosis and progression at 24 months of systemic sclerosis‑associated ILD | Roeser, 2023 (65) | O_3_ | 🗸 |  |  | Pre-ILD diagnosis exposure to O_3_ on severity at diagnosis: O_3_ adjusted OR: 1.12, 95% CI 1.05–1.21; p=0.002; Progression at 24 months: O_3_ adjusted OR: 1.10, 95% CI 1.02–1.19; p =0.02 |
|  |  | PM_2.5_, PM_10_ and NO_2_, |  | 🗸 |  | The severity at diagnosis: PM_2.5_ OR 0.87 (95% CI 0.64–1.20)p=0.39; PM_10_ OR 0.91 (95% CI 0.75–1.11) p=0.36; NO_2_ OR 0.95 (95% CI 0.91–1.00) p=0.08; Progression at 24 months:PM_2.5_ OR0.89 (95% CI 0.75–1.04)p=0.15; PM_10_ OR 0.93 (95% CI 0.83–1.04)p=0.18; NO_2_ OR 0.96 (95% CI 0.10–1.02)p=0.19 |
| Decreased QoL or Respiratory symptoms in Fibrotic Sarcoidosis patients | Pirozzi, 2018 (49) | Short-term PM_2.5_ | Measured by KSQ | Measured by SGRQ or LCQ |  | Per IQR increase in 14-day average PM_2.5_, KSQ general health status, score change -6.60 (95% CI −12.51 to −0.68), and lower KSQ lung specific health status, score change −6.91(95% CI −12.73 to −1.09); SGRQ score change 1.87 (95%CI −1.96 to 5.70) LCQ score change −0.66( 95% CI −2.03 to 0.70) |
|  |  | Short-term O_3_ |  | SGRQ, LCQ, or KSQ. |  | Short-term ozone exposure was not associated with respiratory symptoms measured by SGRQ, LCG, or KSQ |
| Development of ILD in patients with CTDs | Chen, 2020 (52) | O_3_ |  |  | 🗸 | Ozone (O_3_ ) exposure (per 10 ppb) was associated with a decreased ILD risk in patients with CTD (aOR, 0.51; 95%CI, 0.33 to 0.79)(p=0.0024) |
| Global DNAm in patients with IPF | Goobie, 2023 (62) | PM_2.5_ and constituent | 🗸 |  |  | Higher PM_2.5_ 3-month exposures prior to blood collection were associated with higher %5 mC in Simmons (β = 0.02, 95%CI 0.0003–0.05, p = 0.047); Higher exposures to sulphate, nitrate, ammonium, and black carbon constituents were associated with higher %5 mC in multiple models |
| Supplemental O_2_ to maintain SpO2 > 88% during 6MWT in IPF patients | Winterbottom, 2018 (48) | PM_2.5_ | 🗸 |  |  | β-coefficient 1.15 (95% CI,0.03-2.26) p=0.044 |
|  |  | PM_10_ |  | 🗸 |  | β-coefficient 0.15 (95% CI, –0.03 to 0.1) p=0.51 |
| Chronic Respiratory Failure | Mariscal-Aguilar, 2023 (63) | CO, NO_2_, and NOx | 🗸 |  |  | For each 0.1 mg/m³ increase in CO:OR 1.62 (1.11–2.36)(p = 0.01) and OR of 1.84 (1.1–3.06) at 3Month and 6 month prior to the event; Per 10 µg/m³ increase in NO_2_ and NOx : NO_2_ OR 1.65 (1.01–2.66) (p = 0.04) 6month prior to the event; NOx: OR 1.12 (1.01–1.23) (p = 0.03)and OR 1.20 (1.03–1.38) (p = 0.01) at 3 and 6 months of exposure prior to the event. |
| Inflammatory mediator | Tomos, 2021 (56) | O_3_ | IL-4 level |  |  | O_3_ was positively associated with %change of IL-4 (p=0.014) |
|  |  | PM_2.5_, PM_10_, and NO_2_ |  |  | IL-4 and Osteopontin level | PM_2.5_, PM_10_ and NO_2_ were inversely associated with %changes of IL-4 (p=0.003, p=0.003, p=0.032) and osteopontin (p=0.013, p=0.013, p=0.085) |
